# Sensor-Oriented Framework for Underwater Acoustic Signal Classification Using EMD–Wavelet Filtering and Bayesian-Optimized Random Forest

**DOI:** 10.3390/s25175336

**Published:** 2025-08-28

**Authors:** Sergii Babichev, Oleg Yarema, Yevheniy Khomenko, Denys Senchyshen, Bohdan Durnyak

**Affiliations:** 1 Department of Physics, Kherson State University, 73008 Kherson, Ukraine; yekhomenko@ksu.ks.ua (Y.K.); dsenchishen@ksu.ks.ua (D.S.); 2 Department of Informatics, Jan Evangelista Purkyně University in Ústí nad Labem, Pasteurova 3632/15, 400 96 Ústí nad Labem, Czech Republic; 3 Department of Digital Economy and Business Analytics, Ivan Franko National University, 79000 Lviv, Ukraine; oleh.yarema@lnu.edu.ua; 4 Department of Computer Technologies in Publishing and Printing Processes, Printing Art and Media Technologies Institute, Lviv Polytechnic National University, 79000 Lviv, Ukraine; bohdan.v.durnyak@lpnu.ua

**Keywords:** underwater acoustic sensors, ship acoustic signal classification, empirical mode decomposition, wavelet filtering, bayesian optimization, random forest, feature extraction, non-stationary signal processing

## Abstract

Ship acoustic signal classification is essential for vessel identification, underwater navigation, and maritime security. Traditional methods struggle with the non-stationary nature and noise of ship acoustic signals, reducing classification accuracy. To address these challenges, we propose an automated pipeline that integrates Empirical Mode Decomposition (EMD), adaptive wavelet filtering, feature selection, and a Bayesian-optimized Random Forest classifier. The framework begins with EMD-based decomposition, where the most informative Intrinsic Mode Functions (IMFs) are selected using Signal-to-Noise Ratio (SNR) analysis. Wavelet filtering is applied to reduce noise, with optimal wavelet parameters determined via SNR and Stein’s Unbiased Risk Estimate (SURE) criteria. Features extracted from statistical, frequency domain (FFT), and time–frequency (wavelet) metrics are ranked, and the top 11 most important features are selected for classification. A Bayesian-optimized Random Forest classifier is trained using the extracted features, ensuring optimal hyperparameter selection and reducing computational complexity. The classification results are further enhanced using a majority voting strategy, improving the accuracy of the final object identification. The proposed approach demonstrates high accuracy, improved noise suppression, and robust classification performance. The methodology is scalable, computationally efficient, and suitable for real-time maritime applications.

## 1. Introduction

Ship acoustic signal classification plays a critical role in various maritime applications, such as vessel identification, underwater navigation, and maritime security [1,2]. The ability to accurately classify acoustic signals from different types of ships is vital for monitoring traffic, preventing unauthorized activities, and enhancing safety in maritime zones. The challenge lies in the complexity of acoustic signals, which are often non-stationary and contain noise caused by environmental and operational factors [3]. This necessitates advanced signal processing and classification techniques to ensure reliable and accurate performance. With the increasing density of maritime traffic and heightened security concerns, efficient ship classification systems have become indispensable. Traditional methods relying on human expertise or simple frequency analysis often fail to handle the diversity and complexity of ship acoustic signals effectively. The maritime industry’s growing demand for intelligent systems to classify ship signals in real-time underscores the need for advanced methods capable of dealing with noisy and non-stationary data [4,5]. Moreover, these systems must operate under varying environmental conditions, which adds to the difficulty of designing robust solutions. Addressing this problem is essential for maritime domain awareness, especially in congested or strategic areas.

Traditional methods for ship acoustic signal classification include frequency domain analysis, statistical methods, and basic machine learning algorithms [6,7]. Frequency domain analysis, such as Fourier transform-based methods, provides insight into the spectral characteristics of signals but struggles to capture temporal variations in non-stationary signals [8]. Statistical methods often require extensive manual feature engineering, which is time-consuming and prone to inaccuracies. Machine learning techniques, such as Support Vector Machines (SVMs) and k-Nearest Neighbors (k-NN), have been employed to automate classification tasks [9,10]. However, these methods heavily depend on the quality of manually extracted features and cannot handle the full complexity of non-linear relationships in the data. Deep learning models, including Convolutional Neural Networks (CNNs) and Long Short-Term Memory (LSTM) networks, have shown promise in handling such complexities, but they require large labeled datasets for training and are computationally expensive, which limits their applicability in real-time systems [11,12]. Another promising approach involves Empirical Mode Decomposition (EMD) for preprocessing non-stationary signals [13,14]. EMD decomposes signals into intrinsic mode functions (IMFs), allowing for better analysis and denoising. Wavelet filtering is often combined with EMD to further enhance signal quality by removing noise components [15,16]. However, determining optimal wavelet filtering parameters, such as threshold levels and wavelet types, remains a challenge. Current methods rely on trial-and-error or fixed thresholds, which are suboptimal and limit the generalizability of the models.

Recent studies have demonstrated significant advances in ship-radiated signal classification using deep learning and hybrid feature extraction techniques. Yang et al. [17] introduced a time-frequency pipeline based on two-dimensional Adaptive Chirp Mode Decomposition (2D-ACVMD) with texture features derived from Gray-Level Co-occurrence Matrices (GLCM), achieving high accuracy using Transformer-based neural networks. However, Transformer-based systems are data-intensive and computationally demanding, limiting their suitability for embedded or real-time environments. Liu et al. [18] proposed a Residual Attention Convolutional Neural Network (RACNN) utilizing Mel-Frequency Cepstral Coefficients (MFCCs) and attention-enhanced residual blocks. While achieving 99.34% accuracy on the ShipsEar dataset, MFCC-based representations may struggle in low Signal-to-Noise Ratio conditions due to the loss of fine-grained structural information. Similarly, Hong et al. [19] applied SpecAugment—a data augmentation technique for spectrograms—in conjunction with a Residual Network (ResNet18) to enhance robustness. This approach proved effective with limited labeled data but still required careful hyperparameter tuning and significant memory allocation for real-time deployment. Wang et al. [20] designed the Attention-based Multi-branch Network (AMNet), which leverages convolutional attention modules and a multi-branch architecture. Although resilient to noise, the approach imposes high computational costs and lacks interpretability. Qi et al. [21] used a combination of wavelet-auditory features and a residual Convolutional Recurrent Neural Network (CRNN). This method improves classification precision by leveraging domain-specific transformations but requires substantial computational resources. Xu et al. [22] introduced a 3D feature-fusion strategy that integrates Mel spectrogram and cepstral features using Multi-Scale Dilated Convolution (MSDC) and Multi-Scale Channel Attention (MSCA) mechanisms. While this improves temporal–spatial feature representation, such architectures are not easily portable to low-resource or embedded environments. Finally, Cao et al. [23] developed a lightweight MobileNetV3-based model using Generalized Frequency Cepstral Coefficients (GFCCs), offering a practical trade-off between inference speed and accuracy, yet still relying on predefined handcrafted features.

In contrast, our method integrates EMD-based adaptive decomposition, wavelet filtering with hyperparameter tuning guided by SNR and SURE, and Bayesian-optimized Random Forest classification. Unlike deep learning models, it requires no large-scale labeled data and remains fully interpretable. Its modular and computationally efficient architecture ensures adaptability to new signals and environments, which is essential for real-time maritime surveillance. First, acoustic signals are decomposed into IMFs using EMD, capturing both time-domain and frequency-domain characteristics. Noise is mitigated by applying wavelet filtering to selected IMFs, with the parameters optimized using SNR and SURE criteria. This ensures that the filtering process is tailored to each signal’s characteristics, improving the quality of extracted features. After preprocessing, segments of the filtered signals are processed to extract a comprehensive set of features, including statistical, frequency-domain (FFT-based features), and time-frequency (wavelet) features. These features provide a rich representation of the signal, capturing both global and local characteristics. The Random Forest algorithm, optimized using Bayesian optimization techniques, is then employed for classification. Bayesian optimization ensures the selection of optimal Random Forest algorithm hyperparameters to maximize classification accuracy while minimizing overfitting. We also applied five-fold cross-validation during the model training.

Compared to traditional methods, the proposed approach significantly improves classification accuracy and robustness. The entire methodology is structured as a pipeline, automating the stages of signal decomposition, adaptive wavelet filtering, feature extraction, and classification. This pipeline framework ensures a seamless and efficient workflow, enabling real-time processing of acoustic signals. The optimization of wavelet filter parameters through SNR and SURE criteria automates the selection of the most relevant IMFs, further enhancing signal quality. The Bayesian optimization of classifier hyperparameters fine-tunes the model for superior performance, while the structured pipeline design facilitates scalability and adaptability to different datasets and operational environments. The entire methodology is organized as a structured pipeline, automating the processes of signal decomposition, filtering, feature extraction, and classification. This ensures the seamless integration of different processing steps, making the approach scalable and adaptable for real-time applications. Additionally, the pipeline structure enhances efficiency by systematically optimizing wavelet filtering parameters using SNR and SURE criteria and fine-tuning classifier hyperparameters through Bayesian optimization. The experimental evaluation confirms that this pipeline-based approach achieves superior performance in terms of accuracy, computational efficiency, and robustness across diverse ship acoustic signal datasets. The integration of EMD and wavelet filtering reduces the impact of noise, while Bayesian-optimized Random Forest provides an efficient and scalable solution for classification tasks. Experimental results demonstrate that the method achieves high accuracy across diverse datasets and is computationally efficient, making it suitable for real-time applications.

The main contributions of this research are as follows:

- A structured and adaptive pipeline is proposed for ship acoustic signal denoising and classification, ensuring seamless integration of signal decomposition, wavelet-based filtering, feature extraction, and machine learning classification. The pipeline automates IMF selection, wavelet parameter optimization, and classifier hyperparameter tuning, minimizing the need for manual intervention.
- The study employs Signal-to-Noise Ratio (SNR) and Stein’s Unbiased Risk Estimate (SURE) criteria to determine optimal wavelet filtering hyperparameters for each IMF, improving the efficacy of noise reduction while preserving relevant signal components. The impact of different wavelet families (Daubechies, Symlets, Coiflets, and Biorthogonal) is analyzed to determine the best-performing wavelet for ship signal processing.
- A comprehensive feature extraction strategy is implemented, integrating statistical, frequency-domain (FFT-based), and time-frequency (wavelet-based) features to enhance signal characterization. Feature importance analysis identifies the top 11 most relevant features, reducing computational complexity while maintaining high classification accuracy.

## 2. Methods

**Figure 1:**
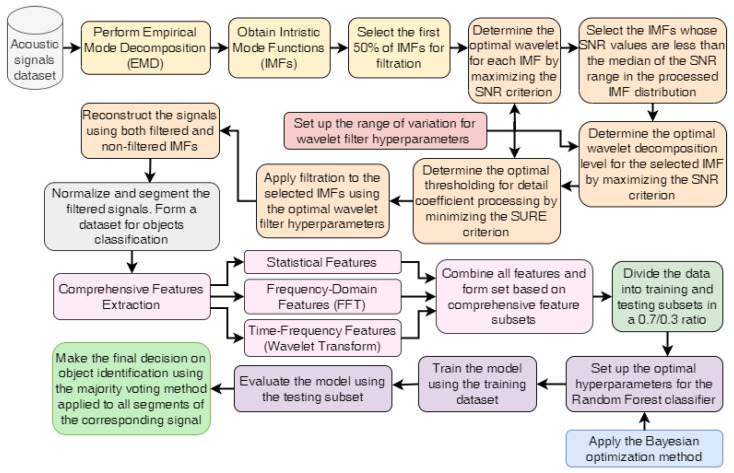
Workflow for acoustic signals processing and objects identification using EMD, adaptive wavelet filtering, and Bayesian-optimized Random Forest algorithm.

Figure 1 presents a comprehensive pipeline for acoustic signal processing and appropriate objects identification, which integrates Empirical Mode Decomposition (EMD), adaptive wavelet filtering, feature extraction, and Bayesian-optimized Random Forest algorithm. The proposed method ensures an automated, structured, and effective approach to handling non-stationary acoustic signals from different types of ships.

Its implementation has the following stages:

I. Signal loading, transforming and decomposition:
  1.1. Transform acoustic signals into numeric ones for the following processing.
  1.2. Implement Empirical Mode Decomposition (EMD) of the raw acoustic signals to obtain Intrinsic Mode Functions (IMFs), which provide a time-adaptive representation of the signal components.
  1.3. Select the first 50% of IMFs for further filtration, as these components typically contain the high-frequency noise components. This proportion was chosen because tests on multiple acoustic signals consistently showed that the early half of the IMFs is sufficient to cover the noise-dominated part, while later IMFs mainly reflect the useful low-frequency structure.
II. Optimization of wavelet filter parameters for each IMFs:
  2.1. Set up the range of wavelet filter hyperparameters variation, including the type of wavelets, the level of wavelet decomposition, and the thresholding coefficient value for processing the detail coefficients.
  2.2. Define the functions used for optimizing the wavelet filter hyperparameters: Signal-to-Noise Ratio (SNR) and Stein’s Unbiased Risk Estimate (SURE).
    - Signal-to-Noise Ratio (SNR) represents the ratio of the power of the true signal to the power of the noise
      (1) $$SNR\;=\;10\cdot log_{10}\left(\frac{\sum x^{2}}{\sum {\left(x-\hat{x}\right)}^{2}}\right)$$
      where *x* is the original signal; $\hat{x}$ is the de-noised signal.
    - Stein’s Unbiased Risk Estimate (SURE) provides an estimate of the mean squared error (MSE) of the threshold process and is useful when the noise follows a Gaussian distribution
      (2) $$SURE\left(\lambda \right)\;=\;\sum_{i=1}^{N}min\left(x_{i}^{2},\lambda^{2}\right)+2\sigma^{2}\sum_{i=1}^{N}1(|x_{i}\left|<\lambda \right)-No^{2}$$
      where $x_{i}$ are the wavelet coefficients; *N* is the number of wavelet coefficients at a specific decomposition level; λ is the threshold; $\sigma^{2}$ is the noise variance; 1(·) is the indicator function.
  2.3. Fix the wavelet decomposition level at 3 and set the threshold coefficient value
    (3) $$\lambda \;=\;C\cdot \sigma \cdot \sqrt{2\cdot logN}$$
    where *C* is a scaling factor that adjusts the strength of threshold (empirically determined as 0.1); *N* is the length of the input data; $\sqrt{2\cdot logN}$ is the universal threshold for optimal de-noising in wavelet shrinkage; σ is the estimated noise level from Median Absolute Deviation (MAD)
    (4) σ=MAD×1.4826
    Here, the constant 1.4826 ensures consistency with the standard deviation if the noise follows a normal distribution.
  2.4. Determine the optimal wavelet function for each IMF by maximizing the SNR criterion.
  2.5. The second phase of IMF selection involves selecting IMFs with a maximum SNR value less than the median of the distribution of all calculated SNRs.
  2.6. Determine the optimal wavelet decomposition level for each selected IMF by also maximizing the SNR criterion.
  2.7. Determine the optimal threshold for detail coefficient processing by minimizing the SURE criterion, ensuring effective noise reduction.
III. Filtration, reconstruction, normalization, and segmentation of signals.
  3.1. Filtrate the IMFs selected in Step 2.5 using the optimal wavelet filter parameters determined for each respective IMF.
  3.2. Reconstruct the signals using filtered and unfiltered IMFs.
  3.1. Perform normalization and segmentation of the filtered signals to prepare a structured data set with appropriate labels to identify the examined objects.
IV. Feature extraction. Three categories of features are extracted from the segmented signals to maximize classification performance:
  4.1. Statistical characteristics (e.g., mean, standard deviation, kurtosis, skewness).
  4.2. Frequency Domain Features using Fast Fourier Transform (FFT):
    - Mean of FFT magnitudes (fft_mean);
    - Standard deviation of FFT magnitudes (fft_std);
    - Energy of FFT magnitudes (fft_energy);
    - Max. FFT magnitude (fft_max);
    - Dominant frequency (fft_dominant_freq)
  4.3. Time-Frequency Features Based on Wavelet Transform:
    - Wavelet mean and std for each level (wavelet_mean_1, wavelet_std_1, etc.)
  4.4. Combine all features. Iterate through all segments and extract features. Combine all extracted features into a comprehensive dataset.
V. Classification procedure implementation.
  5.1. Split the dataset into training and testing subsets in a 0.7/0.3 ratio.
  5.2. Optimize the hyperparameters of the Random Forest classifier using the Bayesian optimization method during model training with five-fold cross-validation.
  5.3. Train the model using optimal hyperparameter values.
  5.4. Evaluate the trained model using the test subset. Analyze the obtained results.
VI. Make the final decision regarding the identification of the objects by applying a majority voting method to all segments of a corresponding signal.

### 2.1. Classification Quality Criteria

To evaluate the accuracy of vessel classification based on segments analysis, the following quality metrics are used:

- Accuracy (ACC): The overall correctness of classification, defined as
  (5) $$ACC\;=\;\frac{TP+TN}{TP+TN+FP+FN}$$
  where TP (True Positives) and TN (True Negatives) represent correctly classified segments, and FP (False Positives) and FN (False Negatives) correspond to misclassified segments.
- F1-score: The harmonic mean of precision and recall, computed as
  (6) $$F1\;=\;2\times \frac{Precision\times Recall}{Precision+Recall}$$
  where:
  (7) $$Precision\;=\;\frac{TP}{TP+FP};\;\;\;\;Recall\;=\;\frac{TP}{TP+FN}$$

For object-level classification, a majority voting method is applied. Each signal is segmented, and the classification results for all segments are aggregated to determine the most frequently predicted class for the entire examined signal. This approach enhances robustness by reducing the impact of individual segment misclassifications and ensuring a more reliable vessel identification outcome.

### 2.2. Final Decision on Object Identification Using the Majority Voting Method

To enhance the reliability of the classification results, a majority voting method was applied for the final decision regarding object identification. This approach aggregates predictions from multiple signal segments, ensuring that the classification of the entire signal is based on the most frequent class assignment. Each ship acoustic signal is segmented into non-overlapping chunks, and each segment was independently classified using the trained Bayesian-optimized Random Forest classifier. Since individual segments may contain varying levels of noise or distinct signal characteristics, direct classification at the segment level may introduce inconsistencies in the final classification of the entire object. To address this issue, the majority voting strategy is implemented to determine the most likely class label for each complete signal.

The decision rule follows a simple but robust approach:

(8) $$C_{final}\;=\;arg\underset{c}{max}\sum_{i=1}^{N_{s}}\delta \left(y_{i},c\right)$$

where $C_{final}$ is the final predicted class for the signal; $y_{i}$ is the predicted class label for segment *i*; $N_{s}$ is the total number of classified segments for the given signal; *c* represents all possible class label; $\delta (y_{i},c)$ is an indicator function that equals 1 if $y_{i}\;=\;c$ and 0 otherwise.

The majority voting mechanism ensures that the final classification is based on the highest number of occurrences of a particular class across all segments, reducing the risk of misclassification caused by local anomalies in individual segments.

## 3. Results

### 3.1. Experimental Data

**Figure 2:**
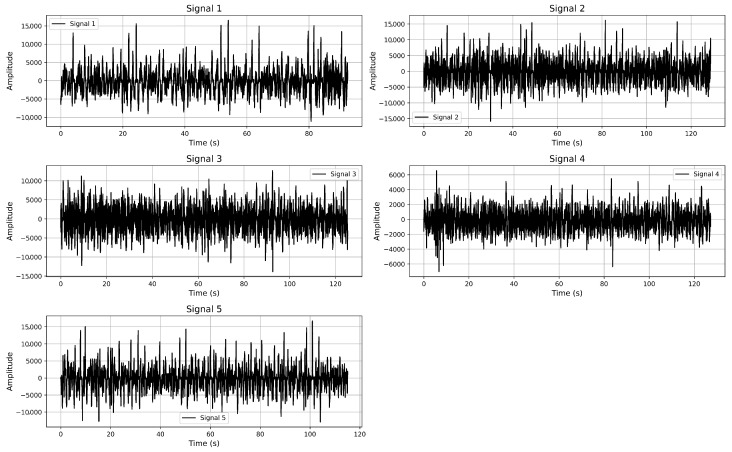
Temporal variability in underwater acoustic signals from different types of vessel.

The acoustic signals were recorded using omnidirectional hydrophones under controlled field conditions in the northwestern region of the Black Sea (Figure 2). Each recording was performed with a sampling rate of 11,025 Hz and a 24-bit resolution using a dual-channel (stereo) setup. The hydrophones were installed at a depth of approximately 15 m and calibrated prior to deployment to ensure precise signal acquisition.

Each signal corresponds to an individual vessel pass and has a duration ranging from approximately 92 to 128 s, providing 1.0 to 1.4 million samples per recording. The recordings were conducted under relatively calm weather conditions to minimize the influence of surface disturbances. Physical parameters, including water temperature (15–18 °C), salinity (16–18 PSU), and depth (15 m), were measured and logged during each recording session. Additionally, GPS coordinates, timestamps, and system configuration details were documented to support reproducibility and enable future comparative studies. The vessels included in this study are as follows:

- Signal 1—Acoustic signal from a border patrol vessel.
- Signal 2—Acoustic signal from another border patrol vessel.
- Signal 3—Signal from a hydrographic survey boat.
- Signal 4—Acoustic recording from a military rocket boat.
- Signal 5—Signal from a reconnaissance ship.

### 3.2. Results of Empirical Mode Decomposition and Signal Filtering

**Figure 3:**
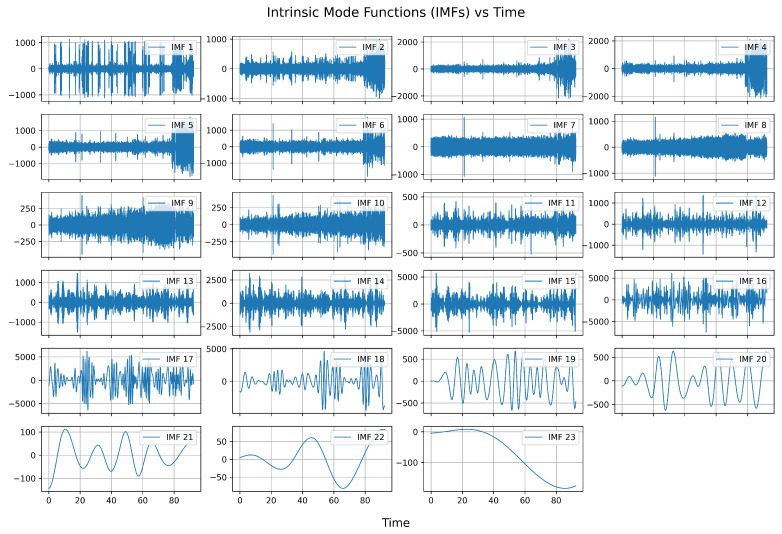
Results of Empirical Mode Decomposition for Signal 1.

The results of the Empirical Mode Decomposition for Signal 1 are presented in Figure 3. Similar results were obtained for other signals.

As can be seen, approximately half of the first IMFs may contain noise. For this reason, in the first step, these IMFs were selected for the filtration procedure.

The orthogonal wavelets Daubechies (db1–db30), symlets, coiflets, and biorthogonal (bior) were used during the simulation procedure. This choice was determined by the following considerations:

- Versatility in Signal Representation: Orthogonal wavelets, such as Daubechies, symlets, and coiflets, offer excellent time-frequency localization, making them well-suited for analyzing non-stationary signals like ship acoustic signals.
- Compact Support and Vanishing Moments: Daubechies wavelets, particularly higher-order ones (db10–db30), provide compact support with an increasing number of vanishing moments, allowing for effective noise reduction and feature extraction.
- Symmetry and Phase Linearity: Symlets and coiflets are modified versions of Daubechies wavelets designed to enhance symmetry, reducing phase distortion in the reconstructed signals, which is crucial for accurate classification.
- Adaptability to Signal Structure: Biorthogonal wavelets (bior) allow for greater flexibility in balancing decomposition and reconstruction properties, making them advantageous for adaptive filtering and denoising.
- Proven Efficiency in Similar Studies: Prior research has demonstrated the efficiency of these wavelet families in applications involving acoustic signal processing, including empirical mode decomposition-based filtering and classification tasks.

**Figure 4:**
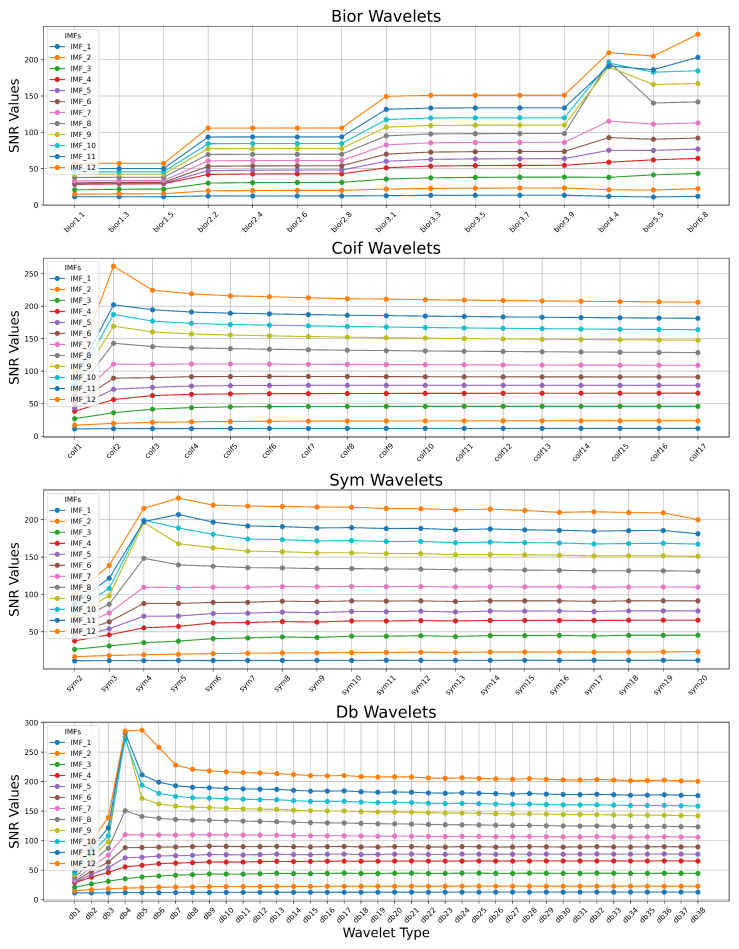
Comparative analysis of the SNR values for different IMFs across four families of wavelets: Biorthogonal (bior), Coiflets (coif), Symlets (sym), and Daubechies (db).

Figure 4 presents a comparative analysis of the SNR values (1) for different IMFs across four families of wavelets: Biorthogonal (bior), Coiflets (coif), Symlets (sym), and Daubechies (db). The SNR values are computed for each IMF after applying wavelet filtering using various wavelet types within each family. Each colored line represents the SNR distribution of a specific IMF across the different wavelet types. The y-axis denotes the SNR values, while the x-axis represents the wavelet type within each family.

The analysis of the obtained results allows us to conclude that within each wavelet family, there exists an appropriate wavelet type that maximizes the SNR criterion when using the same other wavelet filter hyperparameter values. Moreover, the simulation results indicate that the optimal wavelet type varies across different IMFs.

**Figure 5:**
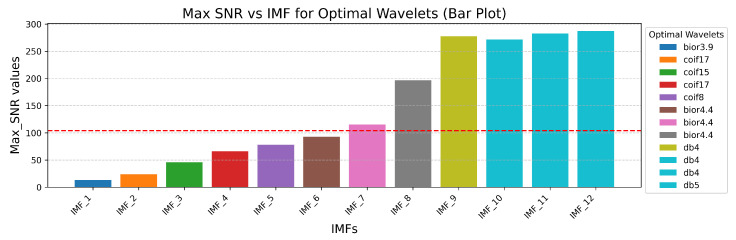
Barplot of the maximal SNR values distribution and the corresponding optimal wavelets for the first 12 IMFs.

Figure 5 presents the chart of the maximal SNR values distribution and the corresponding optimal wavelets for the first 12 IMFs. Further simulation results demonstrated that IMFs with high SNR values do not significantly influence the filtering results. Therefore, the first IMFs with SNR values lower than the median of the entire SNR distribution (represented by the red dashed line) were selected for further processing. The selection of IMFs for wavelet-based filtering was guided by their signal-to-noise characteristics. While initial implementation heuristically targeted the first 50% of IMFs, based on the observation that lower-order IMFs often contain more high-frequency noise, we refined this approach using a data-driven strategy. Specifically, a comparative spectral and SNR analysis was performed across all IMFs. As shown in Figure 5, IMFs with maximal SNR values below the median of the overall SNR distribution were designated as noise-dominant and selected for filtering. This method preserves meaningful high-frequency components while effectively attenuating noise. We acknowledge the potential risk of discarding informative content and have adjusted the description to emphasize that IMF selection is not based on a rigid ordinal cutoff but on empirical evaluation of signal quality. This refinement enhances both the robustness and transparency of the filtering procedure.

**Figure 6:**
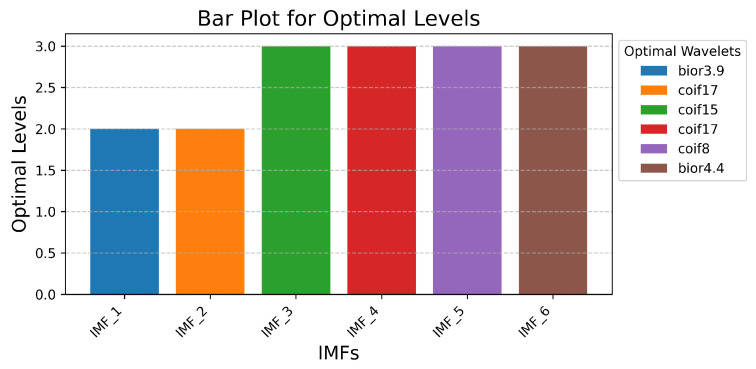
Simulation results for determining the optimal wavelet decomposition levels.

Figure 6 presents the simulation results for determining the optimal wavelet decomposition levels for the IMFs selected in the previous simulation step, considering the optimal wavelet. An analysis of the obtained results allows us to conclude that the maximal SNR value corresponds to decomposition level 2 for IMF 1 and IMF 2, and level 3 for the other IMFs. These values were utilized in the subsequent step of the simulation procedure implementation.

**Figure 7:**
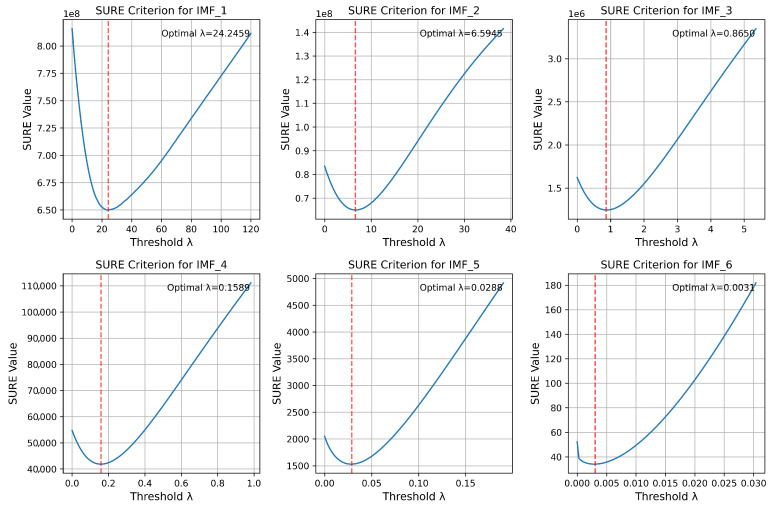
Simulation results for determining the optimal theshold for allocated IMFs.

The next step involves determining the optimal threshold for the soft processing of detail coefficients. As demonstrated by the simulation results, the SNR criterion was not effective in this case, as its value decreased monotonically with an increase in the threshold. Therefore, we employed the SURE criterion (2) to determine the optimal threshold value. The simulation results are presented in Figure 7. As observed, the proposed method enables the determination of the optimal wavelet filter hyperparameters for each IMF.

**Figure 8:**
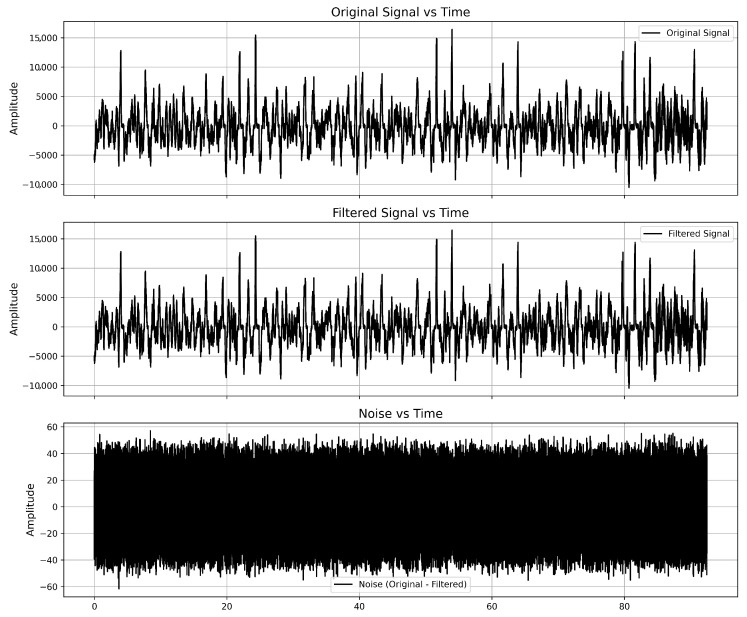
Results of Signal 1 filtration.

Figure 8 illustrates the filtration process applied to Signal 1, demonstrating the effectiveness of the proposed method in allocating and removing noise components.

As we can see, the local particularities of the signal were not changed. The noise distribution appears relatively uniform, suggesting that the filtering process effectively removes random high-frequency components. The amplitude of the noise remains within a significantly lower range compared to the original signal, indicating the effectiveness of the filtering process.

The simulation results enabled us to develop a pipeline for the adaptive wavelet filter application in acoustic signal denoising. Algorithm 1 presents the stepwise procedure for implementing this pipeline.
**Algorithm 1:** Pipeline Filtering Procedure for Acoustic Signal Processing
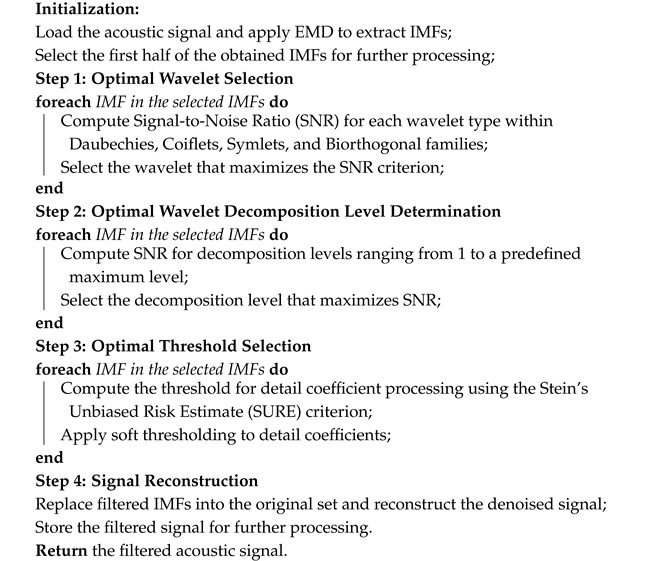


### 3.3. Results of Filtered Signals Classification

In order to evaluate the classification performance of different models, we compared Random Forest (RF), Support Vector Machine (SVM), Gradient Boosting Decision Trees (GBDT), a two-layer Neural Network (NN), and the K-Nearest Neighbors (KNN) algorithm. For each classifier, Bayesian optimization was applied to identify optimal hyperparameter values using five-fold cross-validation. The complete set of tunable hyperparameters and the final selected values are shown in Table 1. Notably, the performance of the SVM with radial basis function (RBF) kernel on our dataset was poor, even after optimization. Changing the kernel to a linear or polynomial variant did not improve results significantly.

**Figure 9:**
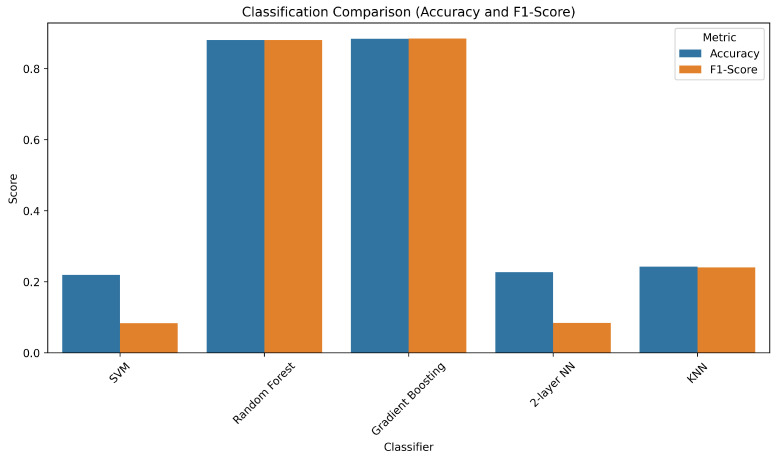
Comparison of classification accuracy and weighted F1-score across different classifiers: SVM (RBF), Random Forest, Gradient Boosting, two-layer Neural Network, and K-Nearest Neighbors (KNN).

Figure 9 presents the classification results for the investigated models. Random Forest and Gradient Boosting classifiers produced the highest accuracy and weighted F1-score values, both exceeding 88% accuracy. Although their results are nearly identical, Random Forest (RF) was selected as the primary classifier in our pipeline due to its inherent resistance to overfitting via bagging, lower sensitivity to hyperparameters, and higher interpretability. Furthermore, RF integrates seamlessly with feature importance ranking mechanisms such as Mean Decrease in Impurity (MDI), enabling effective dimensionality reduction and model simplification.

The selection of a 1000-point segment length was the result of extensive empirical testing aimed at balancing classification performance, model generalization, and computational feasibility. We evaluated multiple segment lengths—500, 750, 1000, 1500, and 2000 points—across several classification models, including Support Vector Machines (SVM), Random Forest (RF), k-Nearest Neighbors (KNN), neural networks (MLP), and deep learning architectures. Direct application of classifiers to raw signal segments yielded unstable and suboptimal performance, primarily due to limited dataset size, segment diversity, and the presence of nonstationary noise in the unstructured inputs. The simulation results suggest that shorter segments (e.g., 500 points) lacked sufficient temporal and spectral resolution, which degraded feature quality and led to increased classification variance. Conversely, longer segments (e.g., 2000 points) introduced redundancy, amplified nonstationary effects, and reduced the number of available training samples per class, critical under small-sample constraints. The 1000-point configuration provided an effective trade-off: it retained informative statistical, spectral (FFT-based), and time-frequency (wavelet-based) patterns, while preserving a sufficient number of training instances for reliable classifier learning and evaluation. This segmentation length also ensured compatibility with the feature extraction pipeline and maintained a high signal-to-noise ratio.

Based on this configuration, 45 features were extracted per segment, encompassing statistical moments, frequency-domain descriptors, and wavelet-based energy measures. Although the full feature set could be used for classification, we employed a feature ranking strategy using the Mean Decrease in Impurity (MDI) criterion derived from the Random Forest model. This allowed us to quantify each feature’s contribution to reducing classification uncertainty and to select a compact subset of the most informative features, thereby minimizing computational complexity while preserving classification accuracy. After ranking the features, we performed a cross-validated empirical evaluation to determine the optimal number of features. Our analysis showed that selecting the top 11 most informative features resulted in the best trade-off between classification accuracy and model efficiency. Adding more features did not lead to significant improvements and, in some cases, even slightly degraded performance due to redundancy or noise amplification. Furthermore, a stepwise elimination process revealed that removing features beyond the 11th position initially had no impact, but progressively reduced classification accuracy.

Thus, the final set of 11 features was selected based on both their MDI ranking and their empirical contribution to cross-validation performance. This dimensionality reduction improves the generalizability of the model while decreasing the model operation time, which is critical for real-time ship signal classification. Moreover, the computational complexity of the pipeline remains moderate due to segmentation and Random Forest efficiency, and the experimental results indicated that as the dataset grows with additional acoustic signals, model performance converges to stable accuracy levels without loss of scalability. The selected features maintain model interpretability and stability, aligning with the core design principles of our pipeline.

**Figure 10:**
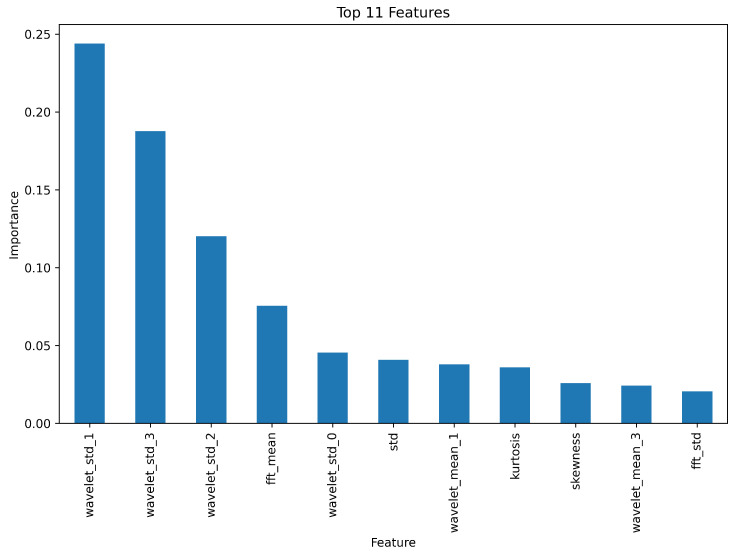
The distribution chart of the importance of the top 11 features when applying the Random Forest algorithm.

**Figure 11:**
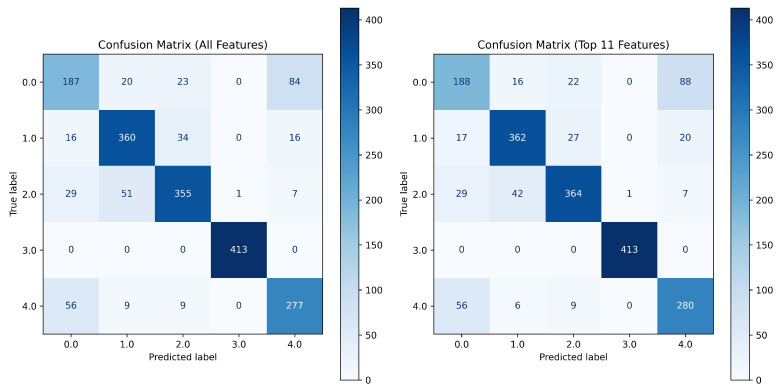
The confusion matrices obtained on the test data when using all features and the selected 11 most important ones.

The step-by-step process for filtering, feature extraction, model training, and evaluation is presented in Algorithm 2. Figure 10 presents the distribution chart of the importance of the top 11 features when applying the Random Forest algorithm. The confusion matrices obtained on the test data when using all features and the selected 11 most important ones are presented in Figure 11. An analysis of these charts confirms the validity of feature extraction based on their importance level, as the number of correctly identified segments is higher when using a limited set of the most important features.

Table 2 presents the segments classification results for all signals. The final results presented in Table 2 demonstrate that applying majority voting (8) improves overall classification accuracy at the object level compared to the direct classification of individual segments. This confirms the effectiveness of aggregated decision-making in ship acoustic signal classification, ensuring a more stable and reliable identification process.
**Algorithm 2:** Classification of Filtered Acoustic Signals Using Optimized Random Forest
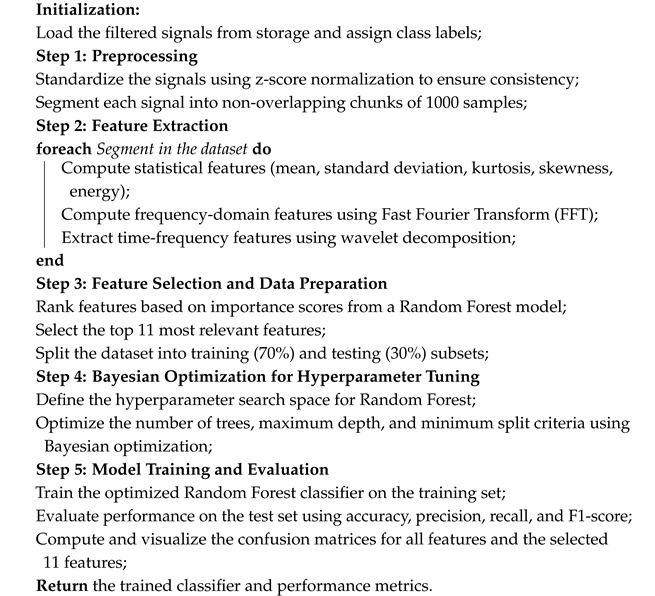


### 3.4. Validation on Synthetic Signals

To evaluate the robustness and generalizability of the proposed framework, we performed simulation-based validation using synthetically augmented signals derived from the original five ship recordings. For each original signal, two variations were generated by introducing controlled distortions such as additive white Gaussian noise, frequency scaling (up to ±5%), random phase shifts, and amplitude modulation. These transformations were designed to mimic realistic environmental conditions, such as changes in vessel speed, sensor position, or underwater propagation effects. The proposed pipeline was applied to these new signals without retraining the model.

Since the classification model was trained on signal segments of 1000 points each, the same segmentation strategy was applied to the synthetic validation signals. Each simulated signal was divided into non-overlapping segments of fixed length (1000 points per segment), consistent with the original training pipeline. This uniform segmentation ensures compatibility with the trained model and allows fair assessment of generalization without retraining. The number of segments per signal was 500.

Each synthetic signal was passed through the entire pipeline: EMD decomposition, adaptive wavelet filtering with SNR/SURE-based parameter optimization, signal reconstruction, normalization, segmentation, multi-domain feature extraction, and final classification using the pre-trained Random Forest model. The results of segment-level classification were aggregated using majority voting to determine the object-level identity.

As shown in Table 3, the proposed framework demonstrated high classification accuracy across all synthetic signals.

Across all synthetic signals, segment-level precision and recall remained above 78%, while the object-level classification accuracy, obtained via majority voting, consistently reached 100%. The overall average accuracy of segment-level classification was approximately 87%, confirming the robustness of the proposed framework against a variety of signal distortions. Furthermore, we observed that variations in the number of segment-level misclassifications had minimal impact on the final object identification, highlighting the stability of the voting-based decision strategy. This finding supports the practical applicability of the method in real-world scenarios where partial signal corruption or variability is expected. These validation outcomes provide further support for the conclusions discussed below.

## 4. Discussion and Conclusions

This work presents an automated and modular framework for ship acoustic signal classification, combining EMD-based decomposition, adaptive wavelet filtering, and a Bayesian-optimized Random Forest classifier. The method effectively addresses challenges posed by nonstationary and noisy signals commonly encountered in underwater sensing environments.

The experimental results demonstrate that the proposed adaptive wavelet filtering strategy significantly enhances signal quality by suppressing noise while preserving relevant information. Feature selection further improves model performance: a compact set of 11 features—drawn from statistical and frequency domains and time–frequency domains—outperforms the full feature set, offering a better trade-off between accuracy and computational efficiency. Confusion matrix analysis confirms the reliability of this reduced feature set.

The integration of SNR and SURE criteria for wavelet parameter optimization enables IMF-specific denoising, improving the robustness of feature extraction. In parallel, Bayesian optimization systematically fine-tunes classifier hyperparameters, enhancing generalization while preventing overfitting.

Compared to recent deep learning approaches such as RACNN [18], ResNet18 with SpecAugment [19], AMNet [20], and Transformer-based 2D-ACVMD [17], our pipeline offers several practical advantages. These neural models, although highly accurate on benchmark datasets, require extensive labeled data and significant computational resources. Our sensor-oriented framework, in contrast, achieves competitive performance with lower overhead and higher interpretability—crucial for real-time embedded maritime applications.

A key innovation lies in the systematic integration of EMD and wavelet filtering, guided by data-driven criteria rather than fixed thresholds. Combined with machine learning optimization, the pipeline achieves accurate classification while remaining computationally lightweight and adaptable. This modularity makes the approach suitable for deployment in sensor networks or onboard acoustic monitoring systems.

Despite strong results, several limitations merit attention. First, the effectiveness of IMF selection and filtering remains partially signal-dependent, potentially affecting generalization to unseen vessel types. Second, the success of the majority voting strategy assumes consistent segment-level predictions, which may degrade under extreme noise or overlapping signals. Third, the Bayesian optimization step, while effective, is computationally intensive and may require acceleration for time-critical tasks.

Validation on synthetically distorted signals (e.g., additive noise, frequency shifts, amplitude modulation) confirmed the robustness of the approach. Without retraining, the pipeline achieved 87% segment-level accuracy and 100% object-level accuracy, underscoring its resilience to signal variability—a valuable attribute for real-world sensor deployments.

While traditional SVMs and deep learning models were explored, they were outperformed by the Random Forest classifier due to the limited size and variability of the dataset. Random Forests offered better generalization, lower overfitting risk, and built-in feature importance evaluation. Bayesian optimization further improved classifier performance, as demonstrated in five-fold cross-validation experiments.

In conclusion, the proposed framework supports intelligent underwater sensing and real-time vessel identification using interpretable, adaptive signal processing. Future work will focus on enhancing generalization through dataset expansion, exploring confidence-weighted voting, and integrating lightweight neural components to further increase portability and scalability.

## Figures and Tables

**Table 1:** Hyperparameter Ranges and Optimized Values for Classifiers.

Classifier	Hyperparameter Range	Optimized Value (s)
SVM (RBF)	*C*: 0.1–10, γ: 0.0001–0.1	C≈6.92, γ≈0.024
Random Forest	*n_estimators*: 50–200, *max_depth*: 1–20, *min_samples_split*: 2–20, *min_samples_leaf*: 1–10	*n_estimators* = 161, *max_depth* = 18, *min_samples_split* = 4, *min_samples_leaf* = 1
Gradient Boosting	*n_estimators*: 50–200, *learning_rate*: 0.01–0.2, *max_depth*: 3–10, *min_samples_split*: 2–20, *min_samples_leaf*: 1–10	*n_estimators* = 165, *learning_rate* ≈ 0.145, *max_depth* = 10, *min_samples_split* = 5, *min_samples_leaf* = 1
2-layer Neural Network	hidden1: 10–100, hidden2: 10–100	hidden1 = 98, hidden2 = 17
KNN	*n_neighbors*: 1–30	*n_neighbors* = 21

**Table 2:** Results of segments classification for the analysed signals.

Signal	Precision	Recall	F1-Score	Correct Cases	Misclassified Cases	Accuracy, %
1	0.65	0.60	0.62	188	126	83
2	0.85	0.85	0.85	362	64	
3	0.86	0.82	0.84	364	79	
4	1.00	1.00	1.00	413	0	
5	0.71	0.80	0.75	280	71	

**Table 3:** Results of model validation for the synthetically augmented signals.

Signal	Precision	Recall	F1-Score	Correct Cases	Misclassified Cases	Accuracy, %
1a	0.83	0.79	0.81	395	105	87
1b	0.87	0.83	0.85	414	86	
2a	0.90	0.88	0.89	439	61	
2b	0.91	0.90	0.91	450	50	
3a	0.86	0.83	0.84	417	83	
3b	0.84	0.80	0.82	400	100	
4a	0.97	0.96	0.96	480	20	
4b	0.99	0.98	0.98	488	12	
5a	0.81	0.84	0.82	420	80	
5b	0.86	0.85	0.85	430	70	

## Data Availability

The datasets generated and analyzed during the current study are publicly available in the GitHub repository at https://github.com/sergiibabichev/Acoustic-Signal-Processing-and-Classification-Pipeline (accessed on 22 August 2025).

## Abbreviations

The following abbreviations are used in this manuscript:

EMD	Empirical Mode Decomposition
IMFs	Intrinsic Mode Functions
SNR	Signal-to-Noise Ratio
SURE	Stein’s Unbiased Risk Estimate
SVM	Support Vector Machines
RF	Random Forest
CNN	Convolutional Neural Network
LSTM	Long Short-Term Memory
2D-ACVMD	two-dimensional Adaptive Chirp Mode Decomposition
